# Supplementary material for: Acceptability of PrEP among MSM and transgender communities—Qualitative findings from two metropolitan cities in India
Source: PLoS One. 2023 Feb 22;18(2):e0281984. doi: 10.1371/journal.pone.0281984 (PMC9946201; doi:10.1371/journal.pone.0281984)
Supplement: S1 File — (DOCX) [file pone.0281984.s001.docx]

**About the MSM and *transgender communities* in India**

This study involves different sub-typologies of MSM and the transgender communities mainly due to differences in sexual behavior and risk patterns. The sub-typologies in both MSM and transgender community groups are elaborated below.

***Transgender communities***

The transgender communities were broadly categorized as 1) Trans-Gender Hijras and 2) Trans-Gender Non-Hijras.

Hijras are a distinct gender found throughout India^[[1]](#footnote-1)^^[[2]](#footnote-2)^. They often identify as women and wear sarees and other clothing reserved for females. Hijras differ from crossdressers, men who wear women’s clothing in that they identify as female, as they feel that they are out of place in a male body. Some hijras participate in the practice of castration to completely severe ties with their male identity. The recognition and reverence of Hijras predate in both pre and post-independent India^[[3]](#footnote-3)^. In the past and currently, Hijras are often invited to joyous occasions like weddings, and baby showers to shower blessings and provide entertainment. While Hijras were originally born into this category by being intersex or exhibiting traits, behaviors, or yearnings of the opposite sex, Hijras are currently identified by their induction as a chela (new inductee), into a Gharana’s (clans) and tutorship under a Nayak (guru)^[[4]](#footnote-4)^. Although Hijras have been recognized in Indian culture for centuries, they did not receive an acknowledgment from legislators until 2014.

While the Trans-Gender Non-Hijras is not a usual category of MSM typologies, this study included trans-sexuals who did not identify as hijras. These participants differ from Trans Hijras for 2 key reasons; they do not train under a Nayak or belong to a Gharana, and they only wear women’s clothing under certain circumstances. While they are not considered Hijra, they are still trans-sexual men who feel out of place in a male body. Their engagement in sexual activity with men makes them an important MSM typology to understand, to gather data, to construct an inclusive and effective HIV intervention for MSM and transgender communities^[[5]](#footnote-5)^.

***Men having Sex with Men (MSM)***

In terms of MSM typology, it is important to note that both Indian and western terminology was used when participants self-identified their sexuality in this study. Despite their sexual preference many Indian MSM do not consider themselves gay or bisexual. These are foreign, western words that do not fully capture the complex nature of their sexuality. As such, Indian MSM often rebukes the labels of gay or bisexual in lieu of their own labels. Figures from several studies have estimated that 30-60% of MSM are married to women^[[6]](#footnote-6)^^[[7]](#footnote-7)^. A thorough history and definition of each of the typologies included in the study are provided below^[[8]](#footnote-8)^.

Double deckers, sometimes referred to as DDs, are MSM who are either the receptive or the insertive partner during sexual intercourse. Little is known about this particular MSM typology. Kothis, for the sake of this study, is an MSM typology. In some cases, Kothis are considered transgender communities because of their effeminate qualities. While these MSM may cross-dress from time, Kothi participants in this study did not identify as trans-gendered and are categorized as cis-gendered MSM. Kothis often embody feminine traits and are always the receptive sexual partners in MSM relationships. They are often the complementary partners of Panthis. Kothis are often at an economic disadvantage and sometimes engage in sex work to meet their financial needs. Panthis are cis-gendered MSM who have masculine presentations. They are often the insertive partner during coitus and are the complementary partners for Kothis. It is important to note that Panthis don’t usually classify themselves as such and are labeled by their partners, either Kothis or transgender communities. Panthis present as hetero-normative males and are indistinguishable from most men^[[9]](#footnote-9)^.

Some participants did identify as either gay or bisexual. These men tended to have a higher socioeconomic status, advanced education, or international exposure. Unlike other Indian MSM, they adopted these terms freely and usually had either the financial, or social freedom to use these labels, without concern for family or social repercussions. Despite their self-identified sexuality as either gay or bisexual, none of these participants openly advertised their sexual preference. Gay are the men who are only attracted to men. There was one gay participant who reported he was married but the usual gay participant is unmarried and does not report having sexual relationships with women. Bisexuals reported having an acknowledged sexual attraction to both men and women.

1. Sharma P. Historical Background and Legal Status of Third Gender in Indian Society; Accessed from <https://web.archive.org/web/20140203031618/http:/www.euroasiapub.org/IJRESS/dec2012/7.pdf>. International Journal of Research in Economics & Social Sciences 2012. [↑](#footnote-ref-1)
2. NACO. Targeted Interventions Under NACP III Operational Guidelines: Accessed from <http://naco.gov.in/sites/default/files/NACP-III.pdf>. 2007. [↑](#footnote-ref-2)
3. Michelraj M. Historical Evolution of transgender Community in India; Accessed from <https://trp.org.in/wp-content/uploads/2015/10/ARSS-Vol.4-No.1-Jan-June-2015-pp.17-19.pdf>. Asian Review of Social Sciences. 2015;ISSN: 2249-6319 Vol. 4 No. 1, 2015, pp. 17-19 ( ). [↑](#footnote-ref-3)
4. Bearak M. Why terms like 'transgender' don't work for India's 'third-gender' communities. Retrieved from <https://www.washingtonpost.com/news/worldviews/wp/2016/04/23/why-terms-like-transgender-dont-work-for-indias-third-gender-communities/?utm_term=.4de54f9ee538>. 2016. [↑](#footnote-ref-4)
5. Dutta A. An Epistemology of Collusion: Hijras, Kothis and the Historical (Dis) Continuity of Gender/Sexual Identities in Eastern India. Gender History Across Epistemologies, 305-329. doi:10.1002/9781118508206.ch142013. [↑](#footnote-ref-5)
6. KHPT. Integrated Behavioural and Biological Assessment: Repeat surveys to assess changes in behaviour and prevalence of HIV/STIs in populations at risk of HIV; Accessed from <http://www.khpt.org/wp-content/uploads/2016/06/Integrated-behavioural-and-biological-assessment.pdf>. 2013. [↑](#footnote-ref-6)
7. Patel VV, Mayer KH, Makadon HJ. Men who have sex with men in India: a diverse population in need of medical attention. Indian J Med Res. 2012;136(4):563-70. [↑](#footnote-ref-7)
8. Solomon SS, Mehta SH, Latimore A, Srikrishnan AK, Celentano DD. The impact of HIV and high-risk behaviours on the wives of married men who have sex with men and injection drug users: implications for HIV prevention. J Int AIDS Soc. 2010;13 Suppl 2:S7. [↑](#footnote-ref-8)
9. Pappas G, Khan, O., Wright, J., Khan, S., & O'Neill, J. . Males who have sex with males (MSM) and HIV/AIDS in India: the hidden epidemic. AIDS Public Policy Journal, 16(1), 4-17. 2001. [↑](#footnote-ref-9)
