# Supplementary material for: Acceptability of PrEP among MSM and transgender communities—Qualitative findings from two metropolitan cities in India
Source: PLoS One. 2023 Feb 22;18(2):e0281984. doi: 10.1371/journal.pone.0281984 (PMC9946201; doi:10.1371/journal.pone.0281984)
Supplement: Attachment — Submitted filename: Reviewer observations on the manuscript ID PONE - Copy - Copy.docx [file pone.0281984.s002.docx]

Reviewer observations on the manuscript ID PONE-D-21-09058

***

Table 1. Reviewer observations in the prescribed template

| S No | Particulars | Reviewer Observations |
| --- | --- | --- |
| 1 | Recommendation (Accept, Minor Revision, Major Revision, Reject) | Major Revision |
| 2 | Is the manuscript technically sound, and do the data support the conclusions? (Answer options: Yes, No, Partly) | Partly |
| 3 | Has the statistical analysis been performed appropriately and rigorously? (Answer options: Yes, No, I don't know, N/A) | N/A |
| 4 | Does the manuscript adhere to the PLOS Data Policy? Additional details can be found at <http://www.plosone.org/static/policies#sharing> |  |
| 5 | Is the manuscript presented in an intelligible fashion and written in standard English? (Answer options: Yes, No) | No |
| 6 | Review Comments to the Author | In table 2 below. |
| 7 | Would you like your identity revealed to the authors of this submission? (Answer options: Yes, No) | No |
| 8 | Do you have any potentially competing interests? If none, type "None." Our policy on competing interests can be found at <http://www.plosone.org/static/policies.action#competing>. | Not to the best of my knowledge |

Table 2. Specific observations

| Section | Line number | Page number (Manuscript) | Comments |
| --- | --- | --- | --- |
| Introduction | 46 | 4 | There are updates in HIV burden estimates in India. The author may like to review, update and reference appropriately. |
|  | 47 | 4 | HIV prevalence is not an indicator to measure the impact of national/global AIDS response. The author might like to relate the impact with new infections, AIDS-related deaths and survival and reference accordingly. |
|  | 58 | 4 | There are updates in HIV burden estimates in India. The author may like to review, update and reference appropriately. |
|  | 60-62 | 4 | The size of MSM population is different from the official estimates of Govt of India. The author may bring that perspective also to provide a more comprehensive picture to the reader. |
| About the MSM and TG population in India | 92-150 | 6-8 | This whole section, while important in itself, appears redundant in the context of this manuscript. The Author may consider including the definition of MSM and TG used in this study in the methodology section and present this whole section about the MSM and TG in India as supplementary material. |
| Methodology | 170 | 9 | The study employed convenience and purposive sampling methods. The author may like to describe the study settings in brief to provide a perspective to the readers. |
|  | 171-172 | 9 | The Author may define the distinct types of MSM and TGs recruited in this study here. |
|  | 175 | 9 | The manuscript not necessarily aims to present any findings by sub-typology of the MSM and TG. The author may consider focusing on the MSM and TG without any further reference to the specific sub-typologies. |
|  | 177 | 10 | May please put the interview guide in brief as a box either in the manuscript or put the detailed tool as supplementary material. |
|  | 187-188 | 10 | Please mention ‘study objectives and methods’ as areas of the training. |
|  | 191-198 | 10 | This paragraph details the study settings. However, it would be good to summarize them here and provide the details in the supplementary material perhaps. |
|  | 222 | 12 | The use of the word ‘paid’ may be reviewed as it breaks the flow of the sentence. |
| Results | 230-595 | 12-30 | 1. The author may consider being a bit more judicious while presenting the quotes and thematic areas. Only the areas which are directly and significantly related to the manuscript objectives may be presented. This will help the manuscript to be more focused and reader-friendly.  2. There is inconsistency in the presentation of the setting and speakers in the quotes. Sometimes, they have been presented by pseudonyms. Sometimes by the respondent ID. Sometimes, the locations have been mentioned. Sometimes, not. Consistency shall be ensured while  the presentation of presenting the setting and speakers in the quotes  3. The results in line 298 appears to be stigmatising a sub-group as inference is being written without mentioning any quote directly referring to the same. At the same time, the findings here doesn't add any significant findings in the context of the manuscript objective. The author may consider removing this result.  4. Consistency shall be established in results in different sections. In line 250, the results say both TG and MSMs had poor knowledge of PrEP. On the other hand, Table 2 specifically mentions as of TGs are well informed on PrEP. The author may like to review the results to ensure consistency in the presentation of results and concluding. |
| Discussion | 631-633 | 32 | 1. The statement that respondents clamoured for PrEP is inconsistent with the study finding of low awareness about PrEP. The author may like to rework the sentences.  2. The manuscript does not present any limitations. It is important to mention the same to provide perspectives to the readers. |
