# Supplementary material for: Acceptability of PrEP among MSM and transgender communities—Qualitative findings from two metropolitan cities in India
Source: PLoS One. 2023 Feb 22;18(2):e0281984. doi: 10.1371/journal.pone.0281984 (PMC9946201; doi:10.1371/journal.pone.0281984)
Supplement: Attachment — Submitted filename: Response to reviweer comments_2nd Aug 2022.docx [file pone.0281984.s003.docx]

**Response to editor comments:**

PONE-D-21-09058R1

Acceptability of PrEP among MSM and Transgender Communities - qualitative findings from two metropolitan cities in India

PLOS ONE

Dear Dr. Ramanaik,

Thank you for submitting your manuscript to PLOS ONE. After careful consideration, we feel that it has merit but does not fully meet PLOS ONE’s publication criteria as it currently stands. Therefore, we invite you to submit a revised version of the manuscript that addresses the points raised during the review process.

Unfortunately, one of the initial reviewers was not available to assess the revisions made, and thus a new reviewer was assigned. This reviewer has very helpfully offered line-by-line suggestions for revising the manuscript. In light of the overall evaluation that the manuscript required professional proofreading in order to be comprehensible to an international readership, following these detailed suggested will be extremely helpful in improving readability. The suggestion about the placement of the limitations, and adding the further limitation indicated, is also in keeping with academic standards. Please when you make these revisions, indicate the location of each revision in the text; this will expedite the editorial review process.

- We are grateful to the editorial team and the reviewers for their constructive feedback. This has greatly helped us to improve the quality of this paper. We have addressed each of the suggestions made by Reviewer 3 have also completed a professional proofreading in alignment with international readership. The limitation paragraph has been moved next to discussion section and all other suggested changes have been incorporated. All changes are made in track-change mode to expedite the review process.

Please submit your revised manuscript by Jul 22 2022 11:59PM. If you will need more time than this to complete your revisions, please reply to this message or contact the journal office at [plosone@plos.org](mailto:plosone@plos.org). When you're ready to submit your revision, log on to <https://www.editorialmanager.com/pone/> and select the 'Submissions Needing Revision' folder to locate your manuscript file.

Please include the following items when submitting your revised manuscript:

- A rebuttal letter that responds to each point raised by the academic editor and reviewer(s). You should upload this letter as a separate file labeled 'Response to Reviewers'.
- A marked-up copy of your manuscript that highlights changes made to the original version. You should upload this as a separate file labeled 'Revised Manuscript with Track Changes'.
- An unmarked version of your revised paper without tracked changes. You should upload this as a separate file labeled 'Manuscript'.
- We have uploaded the above mentioned items as attachments along with the revised manuscript.
- If you would like to make changes to your financial disclosure, please include your updated statement in your cover letter. Guidelines for resubmitting your figure files are available below the reviewer comments at the end of this letter.

  If applicable, we recommend that you deposit your laboratory protocols in protocols.io to enhance the reproducibility of your results. Protocols.io assigns your protocol its own identifier (DOI) so that it can be cited independently in the future. For instructions see: [https://journals.plos.org/plosone/s/submission-guidelines#loc-laboratory-protocols](https://apc01.safelinks.protection.outlook.com/?url=https%3A%2F%2Fjournals.plos.org%2Fplosone%2Fs%2Fsubmission-guidelines%23loc-laboratory-protocols&data=05%7C01%7Csatya%40khpt.org%7C1646d1534bb64aab374808da48a2d62c%7Cfb7b83e72bfe4bb6abf36eece9bb8fe8%7C0%7C0%7C637902161497122952%7CUnknown%7CTWFpbGZsb3d8eyJWIjoiMC4wLjAwMDAiLCJQIjoiV2luMzIiLCJBTiI6Ik1haWwiLCJXVCI6Mn0%3D%7C3000%7C%7C%7C&sdata=iV8MgWHWGP%2BAGqUKRAF4DLIpmohGzA2YuQRSn3zir08%3D&reserved=0). Additionally, PLOS ONE offers an option for publishing peer-reviewed Lab Protocol articles, which describe protocols hosted on protocols.io. Read more information on sharing protocols at [https://plos.org/protocols?utm_medium=editorial-email&utm_source=authorletters&utm_campaign=protocols](https://apc01.safelinks.protection.outlook.com/?url=https%3A%2F%2Fplos.org%2Fprotocols%3Futm_medium%3Deditorial-email%26utm_source%3Dauthorletters%26utm_campaign%3Dprotocols&data=05%7C01%7Csatya%40khpt.org%7C1646d1534bb64aab374808da48a2d62c%7Cfb7b83e72bfe4bb6abf36eece9bb8fe8%7C0%7C0%7C637902161497122952%7CUnknown%7CTWFpbGZsb3d8eyJWIjoiMC4wLjAwMDAiLCJQIjoiV2luMzIiLCJBTiI6Ik1haWwiLCJXVCI6Mn0%3D%7C3000%7C%7C%7C&sdata=%2BYDxVQUDy5gsfcVMlzhM%2BYlDMGES%2BPI0sFa6%2BeLMox4%3D&reserved=0).

  We look forward to receiving your revised manuscript.

Kind regards,

Peter A. Newman, Ph.D
Academic Editor
PLOS ONE

Journal Requirements:

Please review your reference list to ensure that it is complete and correct. If you have cited papers that have been retracted, please include the rationale for doing so in the manuscript text, or remove these references and replace them with relevant current references. Any changes to the reference list should be mentioned in the rebuttal letter that accompanies your revised manuscript. If you need to cite a retracted article, indicate the article’s retracted status in the References list and also include a citation and full reference for the retraction notice.

[Note: HTML markup is below. Please do not edit.]

**Response to reviewers’ comments:**

Reviewer's Responses to Questions

**Comments to the Author**

1. If the authors have adequately addressed your comments raised in a previous round of review and you feel that this manuscript is now acceptable for publication, you may indicate that here to bypass the “Comments to the Author” section, enter your conflict of interest statement in the “Confidential to Editor” section, and submit your "Accept" recommendation.

Reviewer #1: All comments have been addressed

Reviewer #3: (No Response)

2. Is the manuscript technically sound, and do the data support the conclusions?

The manuscript must describe a technically sound piece of scientific research with data that supports the conclusions. Experiments must have been conducted rigorously, with appropriate controls, replication, and sample sizes. The conclusions must be drawn appropriately based on the data presented.

Reviewer #1: Yes

Reviewer #3: Yes

3. Has the statistical analysis been performed appropriately and rigorously?

Reviewer #1: N/A

Reviewer #3: N/A

4. Have the authors made all data underlying the findings in their manuscript fully available?

The [PLOS Data policy](https://apc01.safelinks.protection.outlook.com/?url=http%3A%2F%2Fwww.plosone.org%2Fstatic%2Fpolicies.action%23sharing&data=05%7C01%7Csatya%40khpt.org%7C1646d1534bb64aab374808da48a2d62c%7Cfb7b83e72bfe4bb6abf36eece9bb8fe8%7C0%7C0%7C637902161497122952%7CUnknown%7CTWFpbGZsb3d8eyJWIjoiMC4wLjAwMDAiLCJQIjoiV2luMzIiLCJBTiI6Ik1haWwiLCJXVCI6Mn0%3D%7C3000%7C%7C%7C&sdata=7iU31ARI2uXGggt9k57hXnuUxK50LnnTLndXDkchhGE%3D&reserved=0) requires authors to make all data underlying the findings described in their manuscript fully available without restriction, with rare exception (please refer to the Data Availability Statement in the manuscript PDF file). The data should be provided as part of the manuscript or its supporting information, or deposited to a public repository. For example, in addition to summary statistics, the data points behind means, medians and variance measures should be available. If there are restrictions on publicly sharing data—e.g. participant privacy or use of data from a third party—those must be specified.

Reviewer #1: No

Reviewer #3: Yes

5. Is the manuscript presented in an intelligible fashion and written in standard English?

PLOS ONE does not copyedit accepted manuscripts, so the language in submitted articles must be clear, correct, and unambiguous. Any typographical or grammatical errors should be corrected at revision, so please note any specific errors here.

Reviewer #1: Yes

Reviewer #3: No

6. Review Comments to the Author

Please use the space provided to explain your answers to the questions above. You may also include additional comments for the author, including concerns about dual publication, research ethics, or publication ethics. (Please upload your review as an attachment if it exceeds 20,000 characters)

**Reviewer #1:** (No Response)

- Thank you for your valuable feedback and the time spent to review this paper

**Reviewer #3:** This paper provides qualitative data on the acceptability of PrEP in a diverse cohort of MSM (including those who have a westernised gay identity and those who are described by traditional Indian terms) and transgender women in two different settings in India. The participants in this study do not have access to PrEP, so they are responding to and intervention that has been described to them, rather than one they have personally used.
While I recognise that this paper has already undergone a round of review, I am recommending further revisions which I hope will add to the clarity of the paper and situate its findings more clearly in relation to other literature.

- Thanks very much for your valuable feedback and the comments. We have tried our best to respond to all your comments.

Please note that all the line numbers I reference are from the tracked changes version.
1. Firstly, I recommend professional copy editing for this manuscript, as there are numerous occasions where the wording choice is slightly jarring or the tense wrong. That said, I recognise that this is not always possible so I will endeavour to point out examples of wording choices and tenses.

- Your feedback on the language is very helpful. We have addressed your feedback on the language and have also completed a professional proof-reading for language proficiency.

2. I would strongly suggest that the umbrella term for the transgender respondents be changed to ‘transgender women’ (no capital ‘T’ needed for transgender). The use of ‘people’ initially made me think that both trans men and women – and possibly non-binary people – were included in the study, whereas it is all people with feminine identification (some who identify with the traditional term, hijra., and some who do not). If the authors feel it is inappropriate to use the term ‘women’ I bow to their superior understanding of the cultural context, but in my country when a trans person identifies as a woman it is considered respectful to call her a woman, regardless of what medical interventions she has or has not had.

- **We have used the terminology ‘transgender community’ or ‘transgender communities’ or transgender individuals which is the currently accepted terminology at an international level. We think this is more appropriate and acceptable in the local context. We would prefer to use this terminology instead of the term specifically referring to the community as ‘women’ or ‘men’.**

3. Line 24, suggest ‘involving’ rather than ‘covering

- **Agree. We have incorporated this change.**

4. Line 25 suggest ‘explored’ not ‘tired to explore

- **Agree. We have incorporated this change**

5. Line 32 – no semi colon after ‘acceptance’ – use comma

- **Agree. We have incorporated this change**

6. Line 39 – cut ‘makes a unique attempt to’ instead say ‘provides’

- **Agree. We have incorporated this change**

7. Line 42 – there; s a hanging ‘s’

- **Agree. We have incorporated this change**

8. Lines 67-70 ‘lesser’ should be less, and this line needs a citation

- **Agree. We have incorporated this change**

9. Line 78 – replace ‘sexual orientation’ with ‘gender identity

- **Agree. We have incorporated this change**

10. Line 172 – cut ‘was’

- **Agree. We have incorporated this change**

11. Line 175 ‘participants’’ – needs apostrophe

- **Agree. We have incorporated this change**

12. Lines 188-90 I suggest removing the phrase ‘typically have more effeminate mannerisms’ altogether, but if the author strongly disagree, then change the word ‘effeminate’ – which is pejorative – to ‘feminine’

- **Agree. We have incorporated this change**

13. Line 191. As I have recommended above, this paper will work better cross culturally if the term ‘transgender women’ is used throughout. At line 191, where there is a definition given, this could include ‘transgender women were reported male at birth but self-identify as women or feminine’

- **Agree. We have used transgender community.**

14. Line 203 – I suggest cutting the line that begins ‘A participatory approach’ – as this is usually understood to mean an approach where there is a stronger partnership between the researcher community and the researchers, from the inception of the project to the dissemination of results. What is described here is a considered approach that aims to ‘give back’ to research participants, which is laudable, but it is different to a participatory approach.

- **Agree. We have incorporated this change**

15. Line 225 no hyphen between two and days

- **Agree. We have incorporated this change**

16. Line 226 ‘included’ not ‘includes’

- **Agree. We have incorporated this change**

17. Line 265 – pseudonyms, not name

- **Agree. We have incorporated this change**

18. Line 266 can delete the line that begins ‘this additional consideration’ – extraneous, this is clear from the preceding line.

- **Agree. We have incorporated this change**

19. Line 302 – replace ‘comprehend’ with ‘misunderstood’

- **Agree. We have incorporated this change**

20. Line 303 – ‘the first introduction to THE CONCEPT OF PrEP’ – add the capitalised words

- **Agree. We have incorporated this change**

21. Line 311 - After providing a briefing on PrEP (add italicised words)

- **Agree. We have incorporated this change**

22. Line 312 – delete ‘have’, making it ‘the participants also’

- **Agree. We have incorporated this change**

23. Line 315 – replace ‘communities’ with ‘respondents’ or ‘participants’

- **Agree. We have incorporated this change**

24. Line 317 – ‘find’ should be ‘found’

- **Agree. We have incorporated this change**

25. Line 326 – The participants express a view that PrEP might reduce the need for HIV testing – was this misapprehension addressed with participants in the information provided, given that frequent testing remains important?

- **Yes. After every session, the misapprehensions and concerns that were raised by the participants were addressed and explained. One of the research team members was a medical doctor. He clarified questions related to HIV testing and other biomedical concerns.**

26. Line 327 – avoid the use of ‘shared’ use ‘reported’ or ‘suggested’

- **Agree. We have incorporated this change**

27. Line 341- text marked deleted – in this section a range of participants talk about sex where the lack of protection is forced by insertive partners, which reveal the profound power disparity between feminine or receptive sex partners and insertive partners. It seems important to me to keep some of this in the paper. I recommend cutting the section on recommendation regarding the general community, (384onwards) but keeping the section that relates to experiences of violence.

- **We agree with your suggestion and have retained the section suggested by you.**
- **The community’s recommendations on providing PrEP to other high-risk communities is relevant in the context of reducing HIV transmission. We consider that this is an important piece of information for policy makers to consider. We have therefore retained this recommendation as well.**

28. Line 455 – Don’t use ‘deviation’ – say ‘diversity’

- **Agree. We have incorporated this change**

29. Line 475 – ‘reported’ not ‘shared’

- **Agree. We have incorporated this change**

30. Line 478 – change ‘deteriorate’ to ‘exacerbate’

- **Agree. We have incorporated this change**

31. Line 487 change ‘are not’ to ‘is not’

- **Agree. We have incorporated this change**

32. Line 537 – it is not necessary to start taking PrEP 6 days before risk exposure (See data on ‘on demand’ PrEP by Molina) – this should be acknowledged in the text somewhere

- **Thank you for sharing this new information. We have used the standard information that was available at the time data was collected.**

33. Line 593 – ‘social pariah’s is a loaded term. How about ‘people of lower social status’ instead?

- **Agree. We have incorporated this change**

34. Line 660 – change ‘categorise’ to ‘distinguish’

- **Agree. We have incorporated this change**

35. Line 695 – rather than ;’inclined towards’ – ‘advocated strongly for’ PrEP

- **Agree. We have incorporated this change**

36. Line 697 – apostrophe in participant’s should be participants’

- **Agree. We have incorporated this change**

37. Line 713 ‘their traveling’ should be ‘the mobile’

- **Agree. We have incorporated this change**

38. Line 716-17 – this line takes the Australian study out of context (I am an author of it). The study was in the context of COVID-related changes and not broadly applicable to this context, excepting that is some instances people prefer to take PrEP medication irregularly, particularly if sex is irregular.

- **Agree. Thank you for this clarification.**

39. The paragraph beginning at line 731 should also address the misconception from the results that PrEP could reduce the need for HIV testing.

- **Agree. We have incorporated this change**

40. Line 745 – replace ‘This’ with ‘Discriminatory attitudes displayed by healthcare providers’

- **Agree. We have incorporated this change**

41. Line 755 ‘raised’ rather than ‘brought forth’

- **Agree. We have incorporated this change**

42. Line 805 – move the limitations to the end of the discussion, and commence with, “this study has some limitations’. After the line, ‘None of the participants have ever used PrEP’ add – ‘and they were dependent upon explanations from the research team to understand how it worked’

- **Agree. We have incorporated this change**

7. PLOS authors have the option to publish the peer review history of their article ([what does this mean?](https://apc01.safelinks.protection.outlook.com/?url=https%3A%2F%2Fjournals.plos.org%2Fplosone%2Fs%2Feditorial-and-peer-review-process%23loc-peer-review-history&data=05%7C01%7Csatya%40khpt.org%7C1646d1534bb64aab374808da48a2d62c%7Cfb7b83e72bfe4bb6abf36eece9bb8fe8%7C0%7C0%7C637902161497122952%7CUnknown%7CTWFpbGZsb3d8eyJWIjoiMC4wLjAwMDAiLCJQIjoiV2luMzIiLCJBTiI6Ik1haWwiLCJXVCI6Mn0%3D%7C3000%7C%7C%7C&sdata=%2F%2B8VQfkZpxGB2xglFzT9zwGFGSPqZ4%2BQ%2BWGRMTwx7Ns%3D&reserved=0)). If published, this will include your full peer review and any attached files.


If you choose “no”, your identity will remain anonymous but your review may still be made public.


**Do you want your identity to be public for this peer review?** For information about this choice, including consent withdrawal, please see our [Privacy Policy](https://apc01.safelinks.protection.outlook.com/?url=https%3A%2F%2Fwww.plos.org%2Fprivacy-policy&data=05%7C01%7Csatya%40khpt.org%7C1646d1534bb64aab374808da48a2d62c%7Cfb7b83e72bfe4bb6abf36eece9bb8fe8%7C0%7C0%7C637902161497122952%7CUnknown%7CTWFpbGZsb3d8eyJWIjoiMC4wLjAwMDAiLCJQIjoiV2luMzIiLCJBTiI6Ik1haWwiLCJXVCI6Mn0%3D%7C3000%7C%7C%7C&sdata=Nh7%2FkrNEZE9HNHNb7FzDubljGJHpObbhdC%2B3gYhrtUI%3D&reserved=0).

Reviewer #1: **Yes:** Pradeep Kumar

Reviewer #3: **Yes:** Bridget Haire
