# Supplementary material for: Acceptability of PrEP among MSM and transgender communities—Qualitative findings from two metropolitan cities in India
Source: PLoS One. 2023 Feb 22;18(2):e0281984. doi: 10.1371/journal.pone.0281984 (PMC9946201; doi:10.1371/journal.pone.0281984)
Supplement: Attachment — Submitted filename: Response to Editor Comments_PLOS ONE_22 Jan 2023.docx [file pone.0281984.s004.docx]

ONE-D-21-09058R2

Acceptability of PrEP among MSM and Transgender Communities - qualitative findings from two metropolitan cities in India

PLOS ONE

Dear Dr. Ramanaik,

Thank you for submitting your manuscript to PLOS ONE. After careful consideration, we feel that it has merit but does not fully meet PLOS ONE’s publication criteria as it currently stands. Therefore, we invite you to submit a revised version of the manuscript that addresses the points raised during the review process.

The authors have largely responded to the reviewer comments; however, in so doing they have introduced new errors into the manuscript that prevent it from being accepted in its current form.

1) The revisions are largely responsive to the reviewers' comments. However, this added statement in the Discussion section is simply not true; plus the citations the authors use in fact do the opposite of what they say. This is more problematic since this overstatement and sweeping characterization of many studies about trans people in India is used to claim the present study as unique. In fact, the authors need to cite these studies when they present their own results, and indicate various factors that have in fact already been identified in the published literature about PrEP use among MSM and among transgender people—not mischaracterize existing studies to claim the uniqueness of their own. 

Strangely, these 2 studies (13, 14) are cited as conflating MSM and trans people; but to the contrary, they demonstrate separate inquiries about PrEP in each population:

“In recent years, there has been an increase in the number of studies on that broach the subject of PrEP acceptability among MSM in India (13, 14). These studies do not distinguish transgender communities from the MSM population.”

13. Chakrapani V, Newman PA, Shunmugam M, Mengle S, Varghese J, Nelson R, et al. Acceptability of HIV Pre-Exposure Prophylaxis (PrEP) and Implementation Challenges Among Men Who Have Sex with Men in India: A Qualitative Investigation. AIDS Patient Care STDS. 2015;29(10):569-77.

14. Chakrapani V, Shunmugam M, Rawat S, Baruah D, Nelson R, Newman PA. Acceptability of HIV Pre-Exposure Prophylaxis Among Transgender Women in India: A Qualitative Investigation. AIDS Patient Care STDS. 2020;34(2):92-8.

Agree. We have incorporated the changes accordingly. These studies cover two different groups and are pioneer studies in investigating about PrEP acceptability in transgender women and MSMs separately in India.

Earlier the statement tried to highlight that often there is ambiguity and over representation of transgender by including MSM, transgender men, transgender women and female sex workers under one umbrella. However, particularly these two studies clearly inquire about two different groups. Changes have been accordingly.

2) The following statement in the abstract is erroneous. The authors claim to address these two populations as distinct, but then report as if HIV prevalence is the same; however, as the authors likely know, this is untrue. The sentence either needs to be re-worded or the different prevalence estimates among these populations indicated.

“Men who have sex with men (MSM) and transgender communities in India have 24-times higher HIV prevalence than the general population.”

Also, this information in the abstract conflicts with what is written in the Introduction:

“HIV prevalence among MSM (2.7%) and transgender community people (3.1%) is about 12 to 14 fold higher than the general population (0.22%) (4)

Both of these statements cannot be correct. 24 times higher? Or 12-14 fold higher?

Agree, have incorporated the correct estimation. It is around 15-17 fold higher. Also, the figures for MSM have been updated in the introduction, referring to the latest data from HIV surveillance report 2021.

<https://www.unaids.org/en/regionscountries/countries/india> (same reference) only numbers updated

3) I accept the authors’ response to Reviewer #3, and their use of the term transgender individual is fine, as is transgender community when not referring to an individual. However, the authors still move back and forth between capitalizing “Transgender” and using lower case “transgender”. Note that MSM is capitalized because it is an acronym; the word transgender should not be capitalized, as correctly indicated by the reviewer. It is now presented randomly in both formats, which is incorrect.

The problem with on and off capitalization of transgender starts in the abstract, but is repeated throughout the manuscript:

For ex.: Line 33: “However, when informed about PrEP, both MSM and Transgender communities expressed their willingness to use PrEP...."

But in the previous sentence, it is correctly written as transgender: “This study found that awareness and use of PrEP were minimal among the MSM and transgender communities.”

Agree, incorporated the changes, made it uniform as ‘transgender communities’ or ‘transgender individual’ as per the context

Then on p. 6: “There are very few studies on the acceptability of PrEP among MSM and transgender in India”

This is written without “individuals” or “communities”, just among “transgender”, which is incorrect.

Agree. We have incorporated the changes, added ‘communities’ while referring to transgender community

4) DISCUSSION: This statement in the first sentence below does not logically correspond to the two following sentences. How can respondents “strongly advocate for PrEP” when “awareness was slight” and “knowledge of PrEP received mixed responses”? At least the first sentence needs to be re-written if you mean that AFTER they learned about PrEP, they were strongly supportive….

Also, there should be no apostrophe after participants in the 3rd sentence.

Agree, we stand corrected and have made the changes.

“Due to persistent risks associated with inconsistent use of condoms, most respondents have advocated strongly for PrEP. Very few among the study population had awareness of PrEP and its uses. Awareness was slightly higher more among the transgender communities. For many of the participants’ the first introduction to PrEP was through this study and knowledge of PrEP received mixed responses."

Additionally, it is important that the authors change this statement to indicate that it is not only due to “inconsistent condom use” that PrEP can be helpful for these communities. This statement implies that all sexual encounters are consensual and all HIV risk is an individual choice, as if to lay blame on individuals. In fact, several studies indicate high rates of sexual violence against both MSM and transgender people in India, and how that impacts on condom use--in addition to depression, alcohol use, etc. It is not merely choosing or not to use a condom. The following article shows that violence victimization, for one, is associated with lower rates of condom use. It also shows separate analyses among MSM and among transgender people, which provides yet another example that does not support the authors' claim that all previous HIV prevention research with transgender people in India fails to distinguish them from MSM. While their claim is true to an extent, they need to tone it down AND indicate examples of work (including the citation below, and references 13 and 14 above) that in fact has differentiated clearly between these two populations:

Venkatesan Chakrapani, Peter A. Newman, Murali Shunmugam, Carmen H. Logie & Miriam Samuel (2017) Syndemics of depression, alcohol use, and victimisation, and their association with HIV-related sexual risk among men who have sex with men and transgender women in India, Global Public Health, 12:2, 250-265, DOI: 10.1080/17441692.2015.1091024

Agree. As correctly pointed out ‘inconsistent condom use’ is only one of the factors but more importantly experiences of violence put the MSM and TG communities at a greater risk of HIV infection. This is added in the text and another research article (Shaw et. Al 2012) has been referred to inform the same, in addition to Chakrapani’s paper (2017).

5) The authors are unclear in their response about having the manuscript professionally proofread by someone fluent in English. While improved, it includes many errors in grammar and style, including formatting of citations. Since this journal does not have a proofreading stage, the authors must take this step themselves. For ex., in the first paragraph of the Introduction:

This sentence is incorrect. The way this is cited is wrong: " As per the recently released (2019) HIV estimation report 2019,..."

Agree. We have corrected the sentence

This sentence is missing a close parenthesis after (FSW … : Key populations including female sex workers (FSW, men who have sex with men (MSM), transgender community and persons who inject drugs (PWID)…

Agree. We have corrected the sentence

These comments present only a few of the instances showing the need for proofreading and corrections. The entire manuscript must be professionally proofread and corrected.

Please submit your revised manuscript by Oct 01 2022 11:59PM. If you will need more time than this to complete your revisions, please reply to this message or contact the journal office at [plosone@plos.org](mailto:plosone@plos.org). When you're ready to submit your revision, log on to <https://www.editorialmanager.com/pone/> and select the 'Submissions Needing Revision' folder to locate your manuscript file.

Please include the following items when submitting your revised manuscript:

- A rebuttal letter that responds to each point raised by the academic editor and reviewer(s). You should upload this letter as a separate file labeled 'Response to Reviewers'.
- A marked-up copy of your manuscript that highlights changes made to the original version. You should upload this as a separate file labeled 'Revised Manuscript with Track Changes'.
- An unmarked version of your revised paper without tracked changes. You should upload this as a separate file labeled 'Manuscript'.

If you would like to make changes to your financial disclosure, please include your updated statement in your cover letter. Guidelines for resubmitting your figure files are available below the reviewer comments at the end of this letter.

If applicable, we recommend that you deposit your laboratory protocols in [protocols.io](http://protocols.io/) to enhance the reproducibility of your results. Protocols.io assigns your protocol its own identifier (DOI) so that it can be cited independently in the future. For instructions see: [https://journals.plos.org/plosone/s/submission-guidelines#loc-laboratory-protocols](https://apc01.safelinks.protection.outlook.com/?url=https%3A%2F%2Fjournals.plos.org%2Fplosone%2Fs%2Fsubmission-guidelines%23loc-laboratory-protocols&data=05%7C01%7Csatya%40khpt.org%7C4464108633a241243ed008da8063a7e9%7Cfb7b83e72bfe4bb6abf36eece9bb8fe8%7C0%7C0%7C637963462774042968%7CUnknown%7CTWFpbGZsb3d8eyJWIjoiMC4wLjAwMDAiLCJQIjoiV2luMzIiLCJBTiI6Ik1haWwiLCJXVCI6Mn0%3D%7C3000%7C%7C%7C&sdata=aiJsrP0Knvjcm5q6nGKEbJJ%2FuhceIzzokSwDiis0jtU%3D&reserved=0). Additionally, PLOS ONE offers an option for publishing peer-reviewed Lab Protocol articles, which describe protocols hosted on [protocols.io](http://protocols.io/). Read more information on sharing protocols at [https://plos.org/protocols?utm_medium=editorial-email&utm_source=authorletters&utm_campaign=protocols](https://apc01.safelinks.protection.outlook.com/?url=https%3A%2F%2Fplos.org%2Fprotocols%3Futm_medium%3Deditorial-email%26utm_source%3Dauthorletters%26utm_campaign%3Dprotocols&data=05%7C01%7Csatya%40khpt.org%7C4464108633a241243ed008da8063a7e9%7Cfb7b83e72bfe4bb6abf36eece9bb8fe8%7C0%7C0%7C637963462774042968%7CUnknown%7CTWFpbGZsb3d8eyJWIjoiMC4wLjAwMDAiLCJQIjoiV2luMzIiLCJBTiI6Ik1haWwiLCJXVCI6Mn0%3D%7C3000%7C%7C%7C&sdata=Je%2FoHVgg2tZTZ8IglUh59vsRvvPz%2Ftp1RcPbrwXBbfs%3D&reserved=0).

We look forward to receiving your revised manuscript.

Kind regards,

Peter A. Newman, Ph.D
Academic Editor
PLOS ONE

Journal Requirements:

Please review your reference list to ensure that it is complete and correct. If you have cited papers that have been retracted, please include the rationale for doing so in the manuscript text, or remove these references and replace them with relevant current references. Any changes to the reference list should be mentioned in the rebuttal letter that accompanies your revised manuscript. If you need to cite a retracted article, indicate the article’s retracted status in the References list and also include a citation and full reference for the retraction notice.

[Note: HTML markup is below. Please do not edit.]

[NOTE: If reviewer comments were submitted as an attachment file, they will be attached to this email and accessible via the submission site. Please log into your account, locate the manuscript record, and check for the action link "View Attachments". If this link does not appear, there are no attachment files.]

While revising your submission, please upload your figure files to the Preflight Analysis and Conversion Engine (PACE) digital diagnostic tool, [https://pacev2.apexcovantage.com/](https://apc01.safelinks.protection.outlook.com/?url=https%3A%2F%2Fpacev2.apexcovantage.com%2F&data=05%7C01%7Csatya%40khpt.org%7C4464108633a241243ed008da8063a7e9%7Cfb7b83e72bfe4bb6abf36eece9bb8fe8%7C0%7C0%7C637963462774042968%7CUnknown%7CTWFpbGZsb3d8eyJWIjoiMC4wLjAwMDAiLCJQIjoiV2luMzIiLCJBTiI6Ik1haWwiLCJXVCI6Mn0%3D%7C3000%7C%7C%7C&sdata=N5Cpw9n%2FIvDyQ74SPWL7dj8Imw7kTjkyxEQWs5EIKzI%3D&reserved=0). PACE helps ensure that figures meet PLOS requirements. To use PACE, you must first register as a user. Registration is free. Then, login and navigate to the UPLOAD tab, where you will find detailed instructions on how to use the tool. If you encounter any issues or have any questions when using PACE, please email PLOS at [figures@plos.org](mailto:figures@plos.org). Please note that Supporting Information files do not need this step.
